# Barriers to Utilization of Antenatal Care Services in Eastern Nepal

**DOI:** 10.3389/fpubh.2015.00197

**Published:** 2015-08-14

**Authors:** Krishna Kumar Deo, Yuba Raj Paudel, Resham Bahadur Khatri, Ravi Kumar Bhaskar, Rajan Paudel, Suresh Mehata, Rajendra Raj Wagle

**Affiliations:** ^1^Karuna Foundation Nepal, Kathmandu, Nepal; ^2^Nepal Health Sector Support Program, Ministry of Health and Population, Kathmandu, Nepal; ^3^Saving Newborn Lives Program, Save the Children, Kathmandu, Nepal; ^4^Department of Community Medicine and Public Health, National Medical College, Birgunj, Nepal; ^5^Department of Community Medicine and Public Health, Institute of Medicine, Tribhuwan University, Kathmandu, Nepal

**Keywords:** four antenatal care visits, socio-cultural factors, utilization, Nepal

## Abstract

**Background:**

World Health Organization recommends at least four pregnancy check-ups for normal pregnancies. Ministry of Health and Population Nepal has introduced various strategies to promote prenatal care and institutional delivery to reduce maternal and child deaths. However, maternal health service utilization is low in some selected socio-economic and ethnic groups. Hence, this study aims to assess barriers to the recommended four antenatal care (4ANC) visits in eastern Nepal.

**Methods:**

A cross-sectional quantitative study was conducted in Sunsari district. A total of 372 randomly selected women who delivered in the last year preceding the survey were interviewed using a semi-structured questionnaire. Bivariate and multivariate logistic regression analysis was carried out to identify barriers associated with 4ANC visits.

**Results:**

More than two-third women (69%) attended at least 4ANC visits. The study revealed that women exposed to media had higher chance of receiving four or more ANC visits with an adjusted odds ratio (aOR = 3.5, 95% CI: 1.2–10.1) in comparison to women who did not. Women from an advantaged ethnic group had more chance of having 4ANC visits than respondents from a disadvantaged ethnic group (aOR = 2.4, 95% CI: 2.1–6.9). Similarly, women having a higher level of autonomy were nearly three times more likely (aOR = 2.9, 95% CI: 1.5–5.6) and richer women were twice (aOR = 2.3, 95% CI: 1.1–5.3) as likely to have at least 4ANC visits compared to women who had a lower level of autonomy and were economically poor.

**Conclusion:**

Being from disadvantaged ethnicity, lower women’s autonomy, poor knowledge of maternal health service and incentive upon completion of ANC, less media exposure related to maternal health service, and lower wealth rank were significantly associated with fewer than the recommended 4ANC visits. Thus, maternal health programs need to address such socio-cultural barriers for effective health care utilization.

## Introduction

Evidence suggests that most maternal deaths and pregnancy-related complications can be prevented if pregnant women have access to good quality maternal health services, which include focused care during pregnancy, care around delivery, and immediate post-partum care (1–3). For women with normal pregnancies, World Health Organization (WHO) recommends a minimum of four antenatal care (ANC) visits, ideally at 16, 24–28, 32, and 36 weeks (4). ANC offers an important opportunity for healthcare providers to detect, treat, and prevent pregnancy-related problems and many life-threatening conditions (5). Further, ANC visit helps in timely referral to the appropriate health facilities; ensures birth preparedness and complication readiness for both normal and obstetric emergencies; and provides tetanus toxoid (TT) immunization, iron tablets, de-worming tablets to all pregnant women, and malaria prophylaxis where necessary (6, 7).

Ministry of Health and Population (MoHP) Nepal has been providing maternal and child health services including ANC check-ups from the health system network extended up to the community level (8). To increase demand for and improve access to maternity services, MOHP launched maternity incentives scheme in 2005 to provide each woman a fixed amount of money to cover transportation costs, based on ecological region (9). Further, 4ANC incentive was introduced in 2009 to promote antenatal services along with institutional delivery (9). However, many studies have shown effectiveness of these incentives and thereby utilization of maternal health services has been low by many poor and excluded groups (10–14).

Although substantial improvement was observed over the last decade, Nepal still has high maternal mortality ratio (MMR) compared to many developed countries (15). According to a survey conducted in 2008/09, the MMR in Nepal was estimated to be 229 per 100,000 live births, which accounted for 11% of all deaths among women of reproductive age (15–49 years) (16). Despite ongoing efforts to promote maternal health service utilization, wide disparities prevail among women residing in rural parts and belonging to the lowest wealth quintiles, and from ethnic minorities (17).

Growing body of research suggests that along with supply-side and demand-side factors including individual and household factors, socio-cultural factors, such as women’s autonomy (18), caste/ethnicity, religion and belief (19), women’s media exposure regarding pregnancy and health care (20), influence health seeking behavior. Further, socio-cultural factors are contextual and entrenched (21) and amplify geographical and financial barriers experienced by women to access maternal health services (22). Many studies have documented poor utilization of institutional delivery by Terai’s (Plain region) Muslims and Dalits (23, 24). A qualitative study conducted in Nepal showed that pregnant women’s heavy work-load, women-in-law’s perception of benefit of ANC, her power and control over resources, and relationship between mother-in-law and pregnant women played a vital role in pregnant women’s utilization of ANC (25). However, role of socio-cultural factors including ethnicity, women’s status, education, exposure to media, and others in relation to demographic and economic factors in utilization of antenatal service remain under-investigated in Nepal. Few of the previously conducted studies in Nepal have included women delivered in last 24 months or more as study participants, rendering findings prone to recall bias (26, 27). In addition, since attending 4ANC visits was found to be positively associated with SBA utilization (28), investigating barriers to 4ANC visits will provide evidence for promoting delivery by skilled providers. Therefore, this study aims to identify factors associated with utilization of 4ANC visits among women who delivered within last 12 months of preceding the survey from three Village Development committees (VDC) of Sunsari district.

## Materials and Methods

### Study setting and participants

A cross-sectional quantitative study was conducted in three VDCs: Haripur, Shreepur, and Kushaha of Sunsari district of eastern Nepal. The VDCs are located within 25–32 km south from district headquarters at Inaruwa. The VDCs were selected due to the first author’s familiarity and having a good mix of ethnic groups since the study focused on assessing socio-cultural factors associated with service utilization. Women who had delivered within a 1-year period preceding this study and gave informed consent were interviewed. Sub Health Posts (SHPs), which are the government health institutions within the VDCs, were consulted to get lists of mothers of children born and immunized during last 1 year period. Since, BCG coverage of the selected VDCs was almost 100% of the target provided by Department of Health Services, Nepal, all mothers who delivered in last 12 months are assumed to be included in sampling frame.

### Sampling and sample size

Sample size was calculated by using the formula: *N* = 1.962*pq*/*L*^2^ where *p* is the prevalence of institutional delivery (35.5%) from Nepal Demographic and Health Survey (NDHS) 2011 (17). The final sample size was estimated to be 387 considering 5% allowable error and assuming 10% possible non-response. A list of households was prepared to trace households with mothers of children aged under 1 year. A total of 387 households out of 1064 were selected by systematic random sampling method. A total of 15 mothers could not be met during household visit; hence, 372 mothers were included in current analysis.

### Data collection

Pretesting of questionnaire was carried out in households of adjoining VDC, and necessary modifications were made. The questionnaire was originally prepared in English then translated into the local language of the study area, i.e., Maithili. Enumerators were trained before data collection. Data collection was carried out from July to August 2012. From these households, women who delivered within a period of 1 year preceding this study were interviewed through a household visit using a structured questionnaire. In non-response cases, mother from another immediate household was interviewed. The first author himself was involved in data collection process and supervision of enumerators.

Data checking, editing, and coding were done by the researcher each day. Data entry was done in Epi Data 3, and analysis was carried out in Statistical Package for Social Science (SPSS) 17.

### Outcome variable

Information about 4ANC visits was determined by asking “How many times did you visit a health facility for ANC check-up?” And the number of reported visits was categorized into two categories: women who make at least 4ANC were coded as 1, and women who did not make any visits or made <4 visits were coded as 0.

### Explanatory variables

Altogether, 19 explanatory variables were included in the analysis to assess their association with 4ANC visits based on previous studies in developing countries as well as from researchers’ knowledge on the subject. Most of the selected variables were nominal and few variables, such as wealth quintile, age, women’s autonomy, and knowledge of maternal health services, educational level were categorized into ordinal scales. The variables include socio-demographic and cultural variables including age, caste/ethnicity, religion, education of respondent, education of husband, occupation of respondent, occupation of husband, wealth quintile and women’s autonomy; knowledge, belief, and exposure-related variables including exposure to media, frequency of exposure, contact with Female Community Health Volunteers (FCHVs), frequency of contact with FCHVs, sex of previous child, belief in traditional healer’s practice, knowledge of maternal health services and knowledge of transport incentives on completion of 4ANC, institutional delivery, and first postnatal care (PNC) visit; and biological variables including parity and experience of pregnancy complications in recent pregnancy. Educational level of respondent and her husband was categorized into two category ordinal scale: literate, which includes primary and above qualification and those who can read and write by attending adult literacy class, and illiterate includes never gone to school and unable to read and write.

Similarly, wealth quintile and wealth rank were categorized using principle component analysis of household assets and expenditures. The first and second quartiles were categorized as low (poor), and third and fourth were categorized as high (rich) wealth rank. The ethnicity status of mother was categorized into two categories: disadvantaged includes Dalit and Muslim and relatively advantaged ethnicity includes non-Dalit and advantaged Terai castes, Janjati, and upper caste group. Women’s autonomy was measured by analyzing three key areas: control over finances, decision-making power and the extent to which they have freedom of movement using 10 variables, and households were categorized into three groups using factor analysis from low autonomy to high autonomy. Factor analysis was used to create a female autonomy score, which was divided into terciles. It was further categorized on two ranks, low and high, while carrying out analysis. Exposure to media was categorized into yes or no. The respondents were asked whether they were exposed to any mass media including print, radio, and television depicting information regarding ANC visits and institutional delivery and PNC visits during her last pregnancy.

### Statistical analysis

Bivariate association of independent variables and 4ANC was examined using Chi-square test. A multivariate logistic regression model was developed for the characteristics that were significant in Chi-square test (*p* < 0.1). Stepwise backward elimination process was used in multivariate logistic regression. A *p* value of <0.05 using Chi-square test was considered significant in the final model.

### Research ethics

This study got ethical approval from Institutional Review Board of Institute of Medicine, Maharajgunj Medical Campus, Tribhuwan University, Kathmandu, Nepal. The interviewers explained the research objectives to the participants before starting the interview. Participants were clearly explained about their right to withdraw from the interview at any time. Personal identifiers were removed before analysis.

## Results

### Characteristics of participants

More than three-quarters of the women (77%) were Hindu. Most of the mothers were multiparous (72%), while more than 6% of women became a mother below the age of 20 years. Nearly half (48%) of the respondents were from disadvantaged ethnic group. More than 9 in 10 (94%) women were literate. About 60% mothers had information on availability of free-delivery service, while about 69% mothers had information on transportation incentive on utilization of institutional delivery service. About two in five (37%) mothers had experienced obstetric complications in their recent pregnancy (Tables 1 and 2).

**Table 1:** **Socio-demographic variables and utilization of ANC services**.

Variables	Total *n*(%)^a^	<4ANC (%)	≥4ANC (%)^b^	*p* Value^c^
**Age group of respondents**				
<20	23 (6.2)	26.09	73.91	0.42
20–24	165 (44.3)	34.55	65.45	
25–29	102 (27.4)	33.34	66.66	
30 or above	82 (22.0)	21.36	78.64	
**Caste/ethnicity**				
Disadvantaged	179 (48.1)	46.93	53.07	**0.001**
Relatively advantaged	193 (51.9)	16.06	83.94	
**Religion**				
Hindu	287 (77.1)	24.04	75.96	**0.001**
Muslim	85 (22.9)	54.12	45.88	
**Education of respondent**				
Illiterate	23 (6.2)	43.48	56.52	0.178
Literate	349 (93.9)	30.09	69.91	
**Education of husband**				
Illiterate	38 (10.2)	50	50	**0.007**
Literate	334 (89.8)	28.74	71.26	
**Occupation of respondent**				
Service/business	47 (12.6)	31.91	68.09	0.874
Agriculture/wage labor	325 (87.4)	30.77	69.23	
**Parity**				
Primiparous	103 (27.7)	24.28	75.72	0.08
Multiparous	269 (72.3)	33.46	66.54	
**Occupation of husband**				
Service/business	161 (43.3)	29.81	70.19	0.88
Agriculture/wage	211 (56.7)	31.75	68.25	
**Wealth quintile**				
First (poor)	74 (19.9)	50	50	**0.001**
Second	75 (20.2)	38.67	61.33	
Third	69 (18.5)	28.99	71.01	
Fourth	80 (21.5)	22.5	77.5	
Fifth (rich)	74 (19.9)	14.86	85.14	
**Women’s autonomy**				
Low	125 (33.6)	45.6	54.4	**0.006**
Medium	132 (35.5)	28.79	71.21	
Higher	115 (30.9)	17.39	82.61	
Total	**372**	31.0	69.0	

*^a^Values in parentheses indicate % of total (n = 372)*.

*^b^Indicates row percentage*.

*^c^Indicates Chi-square test of association between 4ANC and other group*.

*Bold font indicates “significantly associated at p < 0.05”*.

**Table 2:** **Knowledge, exposure, and utilization of ANC services**.

Variables	Total *n*(%)^a^	<4ANC (%)	≥4ANC (%)^b^	*p* Value^c^
**Media exposure**				
No	170 (45.7)	47.65	52.35	**0.001**
Yes	202 (54.3)	16.83	83.17	
**Frequency of media**				
At least once a week	185 (49.7)	20.54	79.46	**0.001**
Less than once a week	59 (15.9)	42.37	57.63	
**Contact with FCHVs**				
No	124 (33.3)	47.58	52.42	**0.001**
Yes	248 (66.7)	22.58	77.42	
**Frequency of contact with FCHVs**				
One time	136 (36.6)	31.62	68.38	**<0.001**
Two or more time	114 (30.6)	11.40	88.6	
**Pregnancy/obstetric complication**				
No	233 (62.6)	32.62	67.38	0.357
Yes	139 (37.4)	28.06	71.94	
**Sex of previous child**				
Male	145 (38.9)	33.79	66.21	0.991
Female	148 (39.8)	33.78	66.22	
**Belief in traditional healer’s practices**				
No	140 (37.6)	11.43	88.57	**0.001**
Yes	232 (62.4)	42.67	57.33	
**Knowledge on maternal health services**				
Poor	109 (29.3)	56.88	43.12	**0.001**
Medium	125 (33.6)	35.20	64.8	
High	138 (37.1)	6.52	93.48	
**Knowledge on transportation incentive**				
No	208 (55.9)	47.60	52.4	**<0.001**
Yes	164 (44.1)	9.76	90.24	
Total	372	31.0	69.0	

*^a^Values in parentheses indicate % of total (n = 372)*.

*^b^Indicates row percentage*.

*^c^Indicates Chi-square test of association between 4ANC and other group*.

*Bold font indicates “significantly associated at p < 0.05”*.

### Prevalence of 4ANC coverage

Among 372 respondents, 257 (69%) women received at least ANC visits, while 49 (13%) women received no ANC visits (Table 3). The coverage of 4ANC among women from advantaged ethnic group was higher (84%) than those from disadvantaged ethnic group (53%). Similarly, women from the richest family were more (85%) likely to attend 4ANC check-ups in comparison to women from poorest economic background (50%). Women who have higher level of autonomy (83%) in comparison to lower level of autonomy (54%), mothers who were exposed to media (83%) had higher likelihood of 4ANC visits compared to those who were not exposed to media (52%). Those who had knowledge on transportation incentive (90%) had higher 4ANC coverage than those who did not have knowledge of incentives (52%) (Tables 1 and 2).

**Table 3:** **utilization of ANC services**.

ANC visit (times)	Number	Percentage
0	49	13.2
1–3	66	17.7
≥4 ANC	257	69.1

### Factors associated to utilization of antenatal care services

In bivariate analysis, a number of variables, such as ethnicity, religion, education status of husband, wealth quintile of the family, women autonomy, media exposure, knowledge on maternal health and transportation incentive, showed statistically significant association in 4ANC service utilization (Tables 1 and 2). Multivariate logistic regression showed that women from advantaged ethnicity (aOR = 2.4, 95% CI: 2.1–6.9), having higher level of autonomy (aOR = 2.9, 95% CI: 1.5–5.7) and women from higher wealth quintile (aOR = 2.3, 95% CI: 1.1–5.3) had higher chance of using at least 4ANC services compared to women who were from disadvantaged ethnic groups, women who have low level of autonomy, and women from poor families, respectively.

Similarly, women with good knowledge of maternal health services (aOR = 5.4, 95% CI: 1.3–23.3) and women exposed to media (aOR = 3.5, 95% CI: 1.2–10.1) were more likely to have four or more ANC visits than the women having poor knowledge and women who were not exposed to media information regarding maternal health care (Table 4). In addition, women who believed in traditional healers were less likely (aOR = 0.05 95% CI: 0.01–0.26) to attend 4ANC service compared to women who did not believe in traditional healers.

**Table 4:** **Multivariate logistic regression analysis of factors associated with 4ANC visits**.

Variables	Crude OR (95% CI)	Adjusted OR (95% CI)
**Caste/ethnicity**		
Disadvantaged	1	1
Relatively advantaged	4.62 (2.84–7.44)	2.43 (2.04–6.92)*
**Women’s autonomy**		
Low	1	1
High	3.12 (2.18–7.2)	2.86 (1.47–5.64)*
**Wealth rank**		
Low (poor)	1	1
High (rich)	2.45 (1.27–4.8)	2.28 (1.06–5.25)*
**Media exposure**		
No	1	1
Yes	4.17 (2.72–7.77)	3.48 (1.20–10.05)*
**Frequency of media exposure**		
At least once a week	1	1
Less than once a week	0.35 (0.18–0.65)	0.75 (0.26–2.16)
**Contact with FCHVs**		
No	1	1
Yes	3.11 (1.75–4.72)	0.72 (0.51–2.14)
**Knowledge on maternal health**		
Poor	1	1
High	18.4 (8.17–41.3)	5.44 (1.26–23.33)*
**Knowledge on incentive upon completion of 4 ANC**		
No	1	1
Yes	8.40 (4.66–15.8)	4.85 (1.45–16.14)*
**Belief in traditional healer’s practices**		
No	1	1
Yes	0.17 (0.09–0.30)	0.05 (0.01–0.26)*

*Statistically significant at p < 0.5 level of confidence and adjusted for all other variables in the table.

## Discussion

This study showed a higher prevalence (69%) of 4ANC visits than other recent national studies (17, 29). However, NDHS 2011 also showed higher proportion of women from eastern Terai region to have received ANC in comparison to women from mountain or hill ecological regions. Furthermore, proportion of literate and young women was high among current study participants compared to participants from earlier studies (17), since educational level was found to be strongly associated with ANC utilization (28). Less distance to nearest health facility (78% households within 30 min distance from nearest health post in rural eastern Terai compared to 62% nationally) (30), and easy access to transportation in Terai region might have resulted in higher coverage of 4ANC in current study. A previous study concluded that <30 min distance from nearest health facility was positively associated with higher coverage of ANC visit (28).

Women from advantaged ethnic groups (84%), rich (85%), and having higher autonomy (83%) status had higher chance of getting 4ANC services. These findings are similar to other studies conducted in developing countries (31, 32). A study conducted in Nepal also showed that women from rich family have three times more chances of having 4ANC visit compared to women from poor family (14). Improvement in women’s status and decision-making power regarding their health in rich and advantaged ethnicity, in comparison to poor and disadvantaged ethnicity, could have contributed to the higher 4ANC visit in these groups. Furthermore, access to financial resources, freedom of movement could have enabled these women to utilize antenatal services frequently (33). Women from advantaged ethnicities and rich families are also more likely to have been exposed to media, such as TV, which was reported to be associated with higher uptake of ANC in a previously conducted study in Nepal (19). In addition, women-in-laws from well-off families are more likely to see benefits of utilizing ANC care and encourage daughter-in-laws to utilize it (25). Higher autonomy through better decision-making power might have enabled pregnant women to complete ANC visits even by resisting discouragement from older women and mother in-laws (34). A previous study conducted in Nepal (35) showed women’s higher autonomy to be associated with higher decision-making power including decision on health care use.

In our study, wealth rank was found to be associated with 4ANC visit. Rich women were two times more likely to complete 4ANC visit than poor women. Economic status is linked with access to many services. Moreover, health service is generally linked with cost. Economic status depicted by wealth rank was significantly associated with 4ANC visit. Various studies have tried to depict economic status by various ways (assets possession, amenity scoring, and income), which have produced difficulty in comparison. However, all the research showed that higher economic status is linked with higher maternal health service utilization (36–39).

Women’s knowledge of incentives and health services showed a strong association with women’s utilization of ANC service. Women having good knowledge about maternal health services were more than five times more likely to use ANC services compared to those who had poor knowledge. Similarly, mothers having knowledge on incentive upon completion of 4ANC visit were five times more likely to use 4ANC services than the mothers without such knowledge. Mothers exposed to media were nearly three and half times more likely to complete 4ANC visit than the mothers not exposed to media, similar to the study in Pakistan and Kenya, which showed an association of media exposure with service utilization (40–42). These findings provide support to the conceptual premise that knowledge and information on service sites and available services made them use the available prenatal services. Improvement of health knowledge is important in Nepal especially among disadvantaged communities for two main reasons. First, pregnancy is considered a natural process, and ANC is still considered a curative service and is sought only when problem arises in South Asian culture (43, 44). Second, improving literacy alone is not sufficient to enable them to understand health problem correctly and use proper health services (45). Providing focused and sustained health education through FCHVs, and health workers, therefore, is likely to make women knowledgeable and improve antenatal service utilization (46). A randomized controlled trail conducted in Nepal has previously shown that health education to pregnant women together with their husband was associated with improved health behavior than educating women alone (47).

Women who have faith in traditional healers had less chance of utilizing 4ANC services, which was similar to results from a study conducted in Bangladesh (48). However, it was not clear what proportion of women actually visited traditional healers in their recent pregnancy. Nonetheless, the fact that nearly two-third of current study participants believed in traditional healers’ practices indicates deeply embedded social acceptance to indigenous health system in eastern Terai region of Nepal. Nepal’s health system recognizes both modern and traditional systems of healing (27). It has been shown in Nepal that faith healers can provide health education in a culturally appropriate and cost-effective manner (49). Against this backdrop, involving faith healers in a productive way to provide health education on maternal and child health seems promising.

Current study did not detect an association of age, education of mother, and parity with utilization of 4ANC services, which was contrary to the findings of many studies (14, 36, 37, 50, 51). Although, characteristics of participants, sampling methods, and other factors might have given rise to unexpected findings, it is interesting to note that socio-cultural factors are more strongly associated with 4ANC visit in comparison to demographic and biological characteristics.

Information dissemination through different media, mobilization of FCHVs could increase the use of antenatal services. A study conducted in western Nepal showed that nearly 75% of targeted women were reached through direct and indirect exposure to a radio campaign on family planning. Direct exposure to mass media message and indirect interpersonal communication among women showed a synergistic effect on behavior change (52). Policy makers should concentrate on implementing targeted health mass media campaigns through community groups to bring about small-to-moderate effect on behaviors on the basis of growing global evidence (53). Hence, women’s empowerment through media campaigns could be a game changer for maternal health service utilization. Along with media campaigns, economic upliftment of women and households will help to ease freedom of movement and remove financial barriers to attend ANC. Women from disadvantaged ethnicities, poor, and marginalized families need additional targeting.

This study attempts to explore socio-cultural barriers to ANC utilization through analysis of socio-economic and demographic characteristics of study population. Although findings from the current study are likely to be subjected to recall bias, it can be considered minimum as we included women who delivered in last 12 months. Nonetheless, some limitations of the present study should be acknowledged. The study was carried out in particular setting of the eastern Terai region, which may not be generalizable to communities from hill or mountain regions; and the cross-sectional nature of the study does not allow the making of inferences regarding temporality of association. Further, sample size for current study was calculated using formulae for measuring proportion considering institutional delivery as the primary outcome. However, the calculated sample size has adequate power (>80%) to compare many population groups for association with 4ANC coverage. Many studies have highlighted quality of ANC to be a major barrier to utilization of maternal health care (28, 44); however, this study did not investigate supply-side factors.

## Conclusion

Socio-cultural factors including disadvantaged ethnicity, lack of knowledge of services and exposure to information, lower women’s autonomy, and belief in traditional healers were identified as barriers to 4ANC service utilization in Sunsari district of eastern Nepal. Lower wealth rank was also identified as a strong barrier to 4ANC service utilization. Taken together, socio-cultural factors and wealth rank showed a stronger association with 4ANC service utilization in comparison to demographic and biological factors. Hence, removing socio-cultural and economic barriers can increase 4ANC service utilization in eastern Terai region of Nepal.

## Author Contributions

KD designed the research, pre-tested the tool, carried out the field work, and acquired the data. KD, YP, and RK performed the statistical analysis, conducted literature review, and prepared first draft of manuscript. RB, RP, SM, and RW reviewed the manuscript. SM and RRW supervised the project. All the authors read and agreed on the final version of paper.

## Conflict of Interest Statement

The authors declare that the research was conducted in the absence of any commercial or financial relationships that could be construed as a potential conflict of interest.
